# Lie construction affects information storage under high memory load condition

**DOI:** 10.1371/journal.pone.0181007

**Published:** 2017-07-20

**Authors:** Yuqiu Liu, Chunjie Wang, Haibo Jiang, Hongjian He, Feiyan Chen

**Affiliations:** 1 Department of Psychology and Behavioral Sciences, Zhejiang University, Hangzhou, China; 2 Bio-X Laboratory, Department of Physics, Zhejiang University, Hangzhou, China; 3 Department of Neurology, the Affiliated Hospital of Hangzhou Normal University, Hangzhou, China; 4 Center for Brain Imaging Science and Technology, Key Laboratory for Biomedical Engineering of Ministry of Education, College of Biomedical Engineering and Instrumental Science, Zhejiang University, Hangzhou, China; Xuanwu Hospital, Capital Medical Universty, CHINA

## Abstract

Previous studies indicate that lying consumes cognitive resources, especially working memory (WM) resources. Considering the dual functions that WM might play in lying: holding the truth-related information and turning the truth into lies, the present study examined the relationship between the information storage and processing in the lie construction. To achieve that goal, a deception task based on the old/new recognition paradigm was designed, which could manipulate two levels of WM load (low-load task using 4 items and high-load task using 6 items) during the deception process. The analyses based on the amplitude of the contralateral delay activity (CDA), a proved index of the number of representations being held in WM, showed that the CDA amplitude was lower in the deception process than that in the truth telling process under the high-load condition. In contrast, under the low-load condition, no CDA difference was found between the deception and truth telling processes. Therefore, we deduced that the lie construction and information storage compete for WM resources; when the available WM resources cannot meet this cognitive demand, the WM resources occupied by the information storage would be consumed by the lie construction.

## Introduction

Deception is an intentional attempt to make the receiver believe something that the sender knows is untrue [1–4]. Extensive studies have established that when individuals are trying to conceal a truth, they need to decide how to respond, lying or being honest, to the information contained in a communicative interaction. And this judgment is made based on the truth-related information retrieved from memory [4–7]. If the decision is to deceive, individuals should construct lies based on the truth-related information before they respond [8]. Whereas, if individuals decide to tell the truth, they do not need this construction process, and can give the truthful responses. Thus, researchers have proposed that the deception process demands more attention and memory resources than truth telling [9–12]

The development of technology has boosted deception studies using the techniques of electroencephalogram (EEG), event-related potentials (ERP), and Functional magnetic resonance imaging (fMRI), etc. In the ERP-based deception studies, P3 component is commonly used as an index to reveal the influence of the cognitive process during the deception. The P3 is considered to be a maximal positive-going potential in a time range between 250 and 500 ms after the stimulus onset, and is thought to reflect the processes involved in attention allocation and memory updating [13–15]. It has been found that the effect of deception on the P3 amplitude is different when the studies use different tasks. When the tasks involve the stimulus significance and attention orientation to the truth-related information, such as the guilty knowledge test (GKT), the amplitude of the P3 signal significantly increases by the probe stimulus with the concealed information [16,17]. However, the P3 amplitude decreases when the tasks involves the information processing during the deception [18–22]. In these studies, using an old/new recognition task or the differentiation of deception paradigm, participants need to give appropriate responses according to the instruction. Thus, they have to turn the truth stored in the memory into lies. It makes lying more cognitive demanding than truth telling, and this high workload during the deception process is assumed to suppress the P3 amplitude. Considering that these ERP studies involve both the information storage and processing in the working memory(WM), these results indicate that WM is heavily involved in the deception process. Moreover, fMRI studies also reveal that deception-related brain regions are associated with WM [23–25]. Christ’s group utilized an activation likelihood estimate (ALE) method of meta-analysis and identified the relationship between deception and each part of executive control system [23]. They compared the deception ALE map with the ALE maps of WM, inhibitory control, and task switching; and found that some brain areas which were activated during the deception were implicated to a greater degree in WM than in inhibitory control and task switching. These brain areas included the right prefrontal cortex, inferior parietal cortex, and the junction of the left middle frontal and precentral gyri. Thus, they proposed that WM played an integral role in the deception.

WM is believed to be a system for the “simultaneous processing and storage of information” [26,27]. By this definition, we can infer that WM has the dual functions in the deception process: holding the information of truth and turning the truth into lies. Moreover, it has been also clear that the WM is limited in capacity [28]. Thus, it is interesting to know how WM resources are assigned to the information storage and processing during the deception process. For example, when there is a large amount of information to remember, the information storage demands a lot of WM resources, and the WM is not sufficient for the information processing in deception. Under this circumstance, would the performance of deception be impaired due to the lack of WM resources, or be ensured by taking up the WM resources demanded by information storage? In addition, according to the Activation-Decision-Construction-Action Theory (ADCAT) of deception, deception can be regarded as a top-down process involving four components: activation, decision, construction and action [4,7]. Individuals encode the context of a communicative interaction and activate relevant information from long-term memory in the activation component. In the decision component, according to the retrieval information and the liar’s goals, the individuals decide whether to lie. In the construction component, a lie is constructed on the basis of the truth-related information. Finally, in the action component, liars will deliver the prepared lies to the receiver(s). This theory reveals that the information storage is continuous during the deception, while the information would be transformed into lies in the construction component. According to the ADCAT, the current study focuses on the construction component of deception and aims to figure out the relationship between the information storage and processing.

Moreover, due to the fact that the P3 amplitude is related to both the attention allocation and task load [14,29], previous studies based on P3 component shed little light on the relationship between lying and WM. To solve this problem, a new index for WM capacity, the amplitude of the Contralateral Delay Activity (CDA), is introduced [30–33]. In a visual WM task, participants are presented with a bilateral array of items, and are instructed to remember items in one hemifield. After a retention interval, the memory is tested with a test array. After recording the ERPs while participants performed this task, CDA can be constructed by subtracting the ipsilateral activity from the contralateral activity. Researchers find that the CDA amplitude is directly modulated by the number of items in memory, and can be used as an efficient index for the maintenance of information in memory. Whereas, few research has examined the relationship between the WM and deception based on CDA data.

In addition, in the response period of many previous deception studies, participants are instructed to recognize the stimulus, and then decide how to respond to this stimulus. Considering that deception components are combined with many potential cognitive processes, such as transforming the truth, analyzing the social context, and inhibiting physiological responses [7], the previous ERP results may involve various cognitive processes due to the complex task design. According to the ADCAT, truth-related information is used to make up lies in construction component, which imposes greater cognitive load than truth telling [4,7], and we assume that the construction component is predominantly associated with the WM. Therefore, in this study, we used the CDA as the index to examine the relationship between the WM and the deception construction.

The task in this study was designed based on the old/new recognition paradigm in the visual WM research, and the memory tasks in the deception research [18,34–38]. To avoid the interference of the complex cognitive processes, this study employed a simple memory task. The cue of deception was located at the beginning of each trial, and the participants could know how to respond (deception or truth telling) before the question period. Thus, the test period could be mainly associated with the construction component of deception. In addition, the CDA amplitude was used as the index in this study to exclude the potential interference of other cognitive processes. We assumed that when the participants tried to give a deception response to the question presented, they would suffer a high WM load resulting from the lie construction process. This high WM load might impair the ability to store enough information in WM, and lead to a low CDA amplitude. Thus, we predicted that the response process, deception or truth telling, would modulate the CDA amplitude during the memory test.

## Methods

### Ethical statement

Participants in this experiment signed written informed consent according to the 1964 Declaration of Helsinki at the beginning of the experiment. All procedures were approved and performed in accordance with the guidelines and regulations of the Ethics Committee of Zhejiang University.

### Participants

There are 22 volunteers participated in this experiment (mean age 22 years, s.d. 1.77, 11 females). All these participants had no history of psychiatric or neurological illness, and had normal or corrected to normal vision.

### Procedure

**Fig 1:**
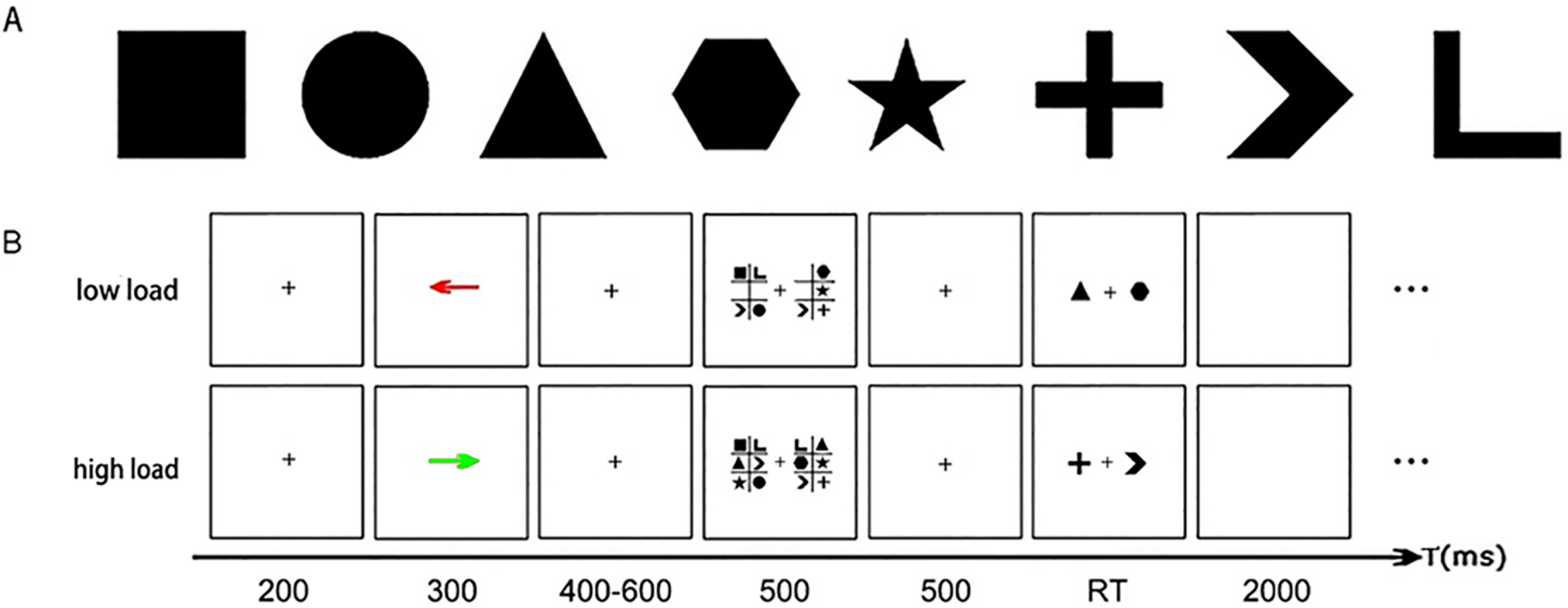
Experimental procedure used in the current study. A) Eight shapes in the stimulus bank. B) The sequence of events in both low-load and high-load trial: in this low-load task, the participants should lie to the stimulus in the left hemifield; in this high-load task, the participants should be honest to the stimulus in the right hemifield.

Participants were seated at a distance of 70 cm from a 17-inch monitor in a sound-attenuated room. The stimulus bank (Fig 1A) comprised eight shapes. All of the stimuli were presented in black against a grey background. Each trial began with the onset of a centrally displayed fixation for 200 ms (Fig 1B). Next, a colored arrow pointing to either the left or right visual hemifield was presented at the center of the screen for 300 ms. Participants were instructed that they should give a deceptive response to the following test period if they saw a red arrow, or give an honest response if they saw a green arrow. This rule was counterbalanced across participants. After a variable delay, which ranged from 400–600 ms, participants were presented with a brief bilateral memory array of the stimuli chosen from the stimulus bank for 500 ms. Participants were instructed to keep their eyes fixated while remembering the items in the cued hemifield. The memory array consisted of 4 or 6 different items in the 2×3 grids presented in each hemifield. Then a 500 ms centrally displayed fixation was inserted and was followed by a test array. In the test period, there was one item appeared in each hemifield. Participants needed to report whether the item in the cued hemifield had been displayed in the memory array, with accuracy rather than response speed being stressed. Participants should respond “yes/no” with the “F” or “J” button. Then there was a 2000 ms blank interval before the next trial. The task consisted of both low- and high-load trials. There were 240 trials under each load condition with a total 480 trials which was presented pseudo-randomly. Before the formal task, there were at least 15 trials for practice to ensure that the participants understood the instructions.

### EEG recording and data analysis

EEG signal was recorded using a 64-channel Quick-scalp with silver chloride electrodes (Neurosoft, Inc. Sterling, USA) and analyzed by a NeuroScan system, Electrodes were located according to the international 10–20 system with a left-mastoid reference. Signal was band-pass filtered from 0.05 to 100 Hz, and sampled at 1000 Hz. Electrode impedances were kept below 10 kΩ. Vertical electrooculogram (VEOG) and horizontal electrooculogram (HEOG) were recorded with two pairs of electrodes, one pair placed above and below the left eye, and another pair placed beside the two eyes. The EEG was referenced offline to the average signal of electrodes on the left and right mastoid. Trials with the amplitude of HEOG exceeding ±50 μV was further removed. The continuous EEG data were segmented into epochs from 200ms before to 2000ms after the memory array onset for all conditions. The 200ms pre-stimulus served as the baseline. The EEG was detrended, and the baseline was corrected. Epochs exceeding the range of −100~100 μV at any channel except HEOG and VEOG were rejected as artifacts. The contralateral waveforms were computed by averaging the activity recorded at right hemisphere electrode sites when subjects were cued to remember the left side of the memory array, with the activity recorded from the right hemisphere electrode sites when they were cued to remember the left side. According to Christ’s research [23], five pairs of electrode sites (F3/F4, FC3/FC4, C3/C4, CP3/CP4, P3/P4) were chosen for the CDA analysis. The CDA was measured by subtracting the ipsilateral activity from the contralateral activity. The averaged CDA waveforms were smoothed by applying a low-pass filter of 30 Hz (zero-phase, 24 dB/octave).

Within each condition, gender differences were compared on all RT and accuracy measures using independent samples t-tests. In no case were significant differences found. Consequently, data were collapsed across gender in subsequent analyses. The behavioral data were analyzed by a repeated measures ANOVA with the task load (4 or 6 items to remember) and response type (deception or truth telling) as two within-subject factors. And the individual’s visual WM capacity was computed using a formula “K = S×(H-F)”[39,40], where K is the number of items stored, S is the number of items in the memory array, H is the hit rate, and F is the false alarm rate. Only the trials with correct responses were used in the ERP analysis. The averaged amplitude of a measurement window of 1200–2000 ms after the onset of the memory array, namely a time window of 200–1000 ms after the onset of the test array, was taken for analysis. And a repeated measures ANOVA was conducted for the CDA amplitude, with the task load, response type and electrode as within-subject factors. In addition, a Bonferroni post-hoc analysis was conducted, if necessary.

## Results

### Behavioral data

**Fig 2:**
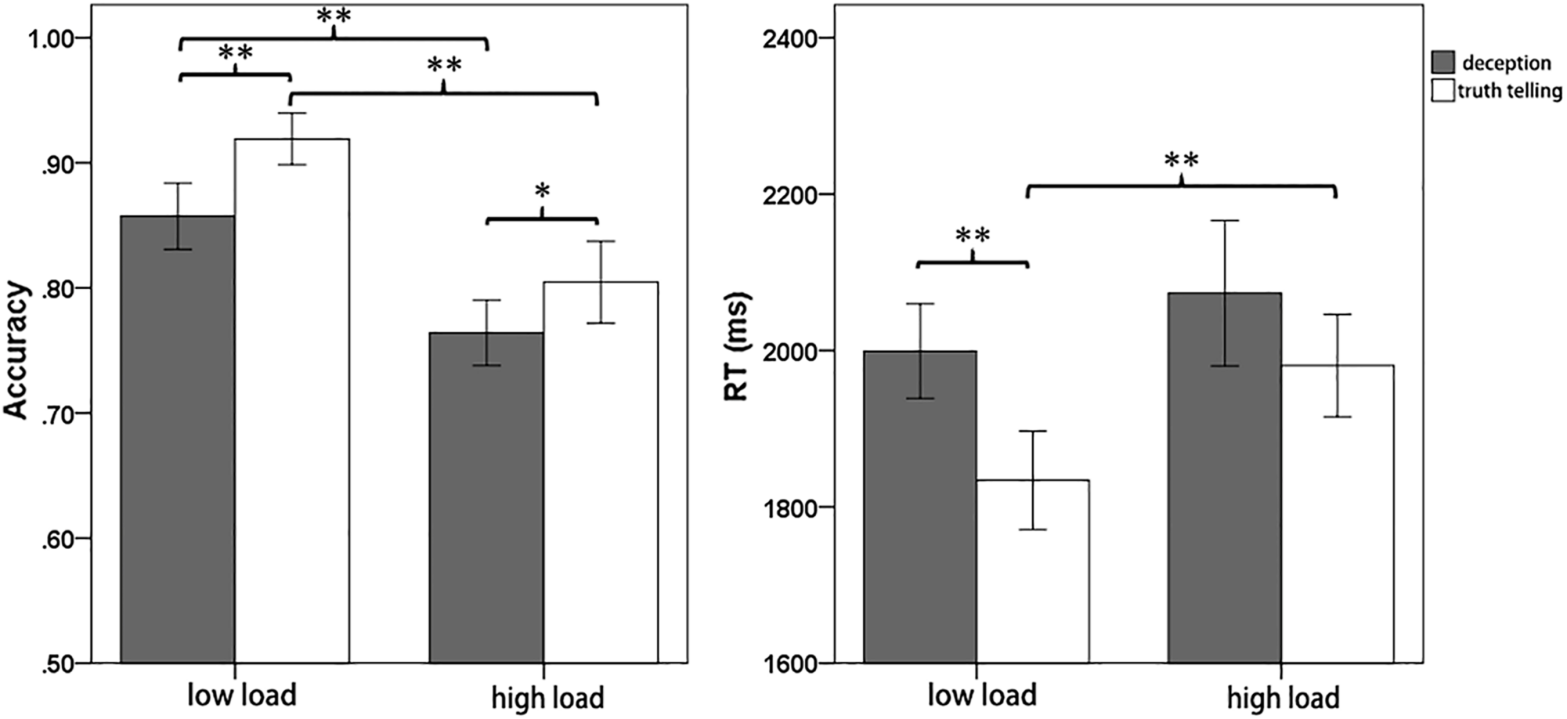
Task performance in this study. Error bar shows one standard error of the mean. **: p < .01; *: p < .05.

The behavioral results of RT and accuracy were shown in Fig 2. The 2 (memory load) × 2 (response type) repeat measures ANOVA on accuracy found significant main effects for both the task load (*F*(1, 21) = 54.26, *p*< 0.01, *partial η*^2^ = 0.72) and response type (*F*(1, 21) = 22.07, *p*< 0.01, *partial η*^2^ = 0.51). Accuracy was lower in the high memory load than the low memory load, and was also lower under the deception condition than the truth telling condition. There was no interaction between memory load and response type. A parallel 2 (memory load) ×2 (response type) repeat measures ANOVA on RT showed similar main effects for the task load (*F*(1, 21) = 8.95, *p*< 0.01, *partial η*^2^ = 0.30) and response type (*F*(1, 21) = 13.79, *p*< 0.01, *partial η*^2^ = 0.40). RTs were longer in the high memory load than in the low memory load, and were longer under the deception condition than under the truth telling condition. There was no interaction between memory load and response type. The results of the WM capacity computation were shown in Table 1. And the 2 × 2 repeat measures ANOVA revealed the main effects for the task load (*F*(1, 21) = 234.94, *p*< 0.01, *partial η*^2^ = 0.92) and response type (*F*(1, 21) = 36.12, *p*< 0.01, *partial η*^2^ = 0.63).

**Table 1:** The results of the WM capacity computation.

WM load	response type	WM capacity	
		means(SD)	95% CI
low	deception	3.41(0.15)	3.34–3.47
	truth telling	3.65(0.13)	3.59–3.71
high	deception	4.10(0.32)	3.96–4.25
	truth telling	4.39(0.21)	4.29–4.48

### ERP data

**Fig 3:**
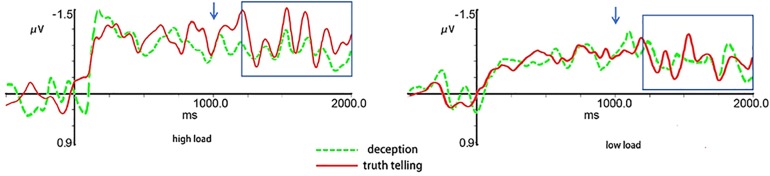
The mean CDA (Contralateral minus Ipsilateral) results for the task conditions. Since the result patterns were similar across the analyzed five electrode pairs, so the figure showed the results on the Fz as the representative. The arrow shows the time when the test array presented, and the time window of average CDA amplitude is also marked.

**Fig 4:**
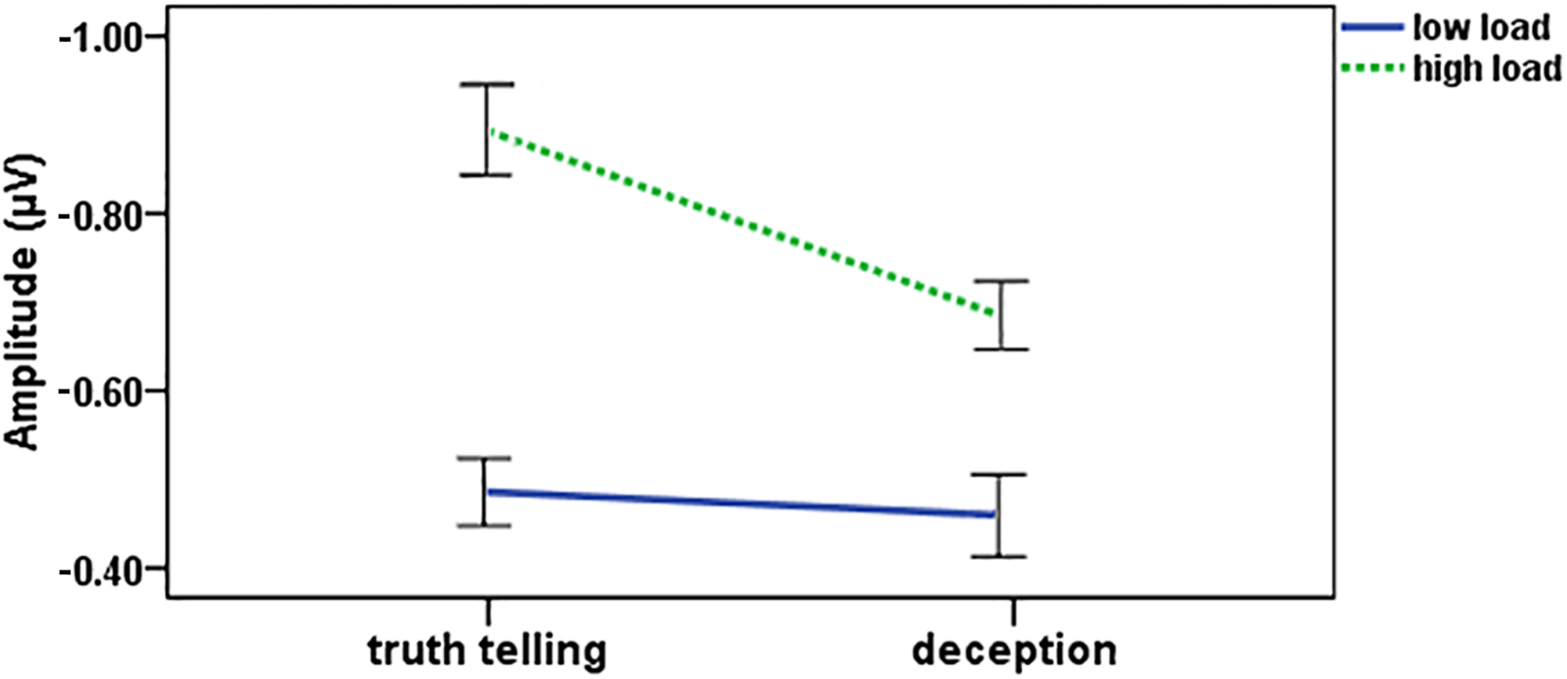
The interaction between the response type and memory load in the CDA analysis. Error bar shows one standard error of the mean.

The ERP results were shown in Fig 3. The 2 (memory load) ×2 (response type) × 5 (electrode) repeat measures ANOVA revealed main effects of response type (*F*(1, 21) = 27.80, *p*< 0.001, *partial η*^2^ = 0.57), memory load (*F*(1, 21) = 10.68, *p*< 0.001, *partial η*^2^ = 0.91), and electrode (*F*(4, 84) = 3.97, *p* = 0.005, *partial η*^2^ = 0.16). There was a significant interaction between response type and memory load (*F*(1, 21) = 7.91, *p* = 0.010, *partial η*^2^ = 0.27) (Fig 4). Post-hoc analysis showed that the CDA amplitude in the high memory load was significantly higher than that in the low memory load under both the deception and truth telling conditions (*p*< 0.001). Whereas, the CDA amplitude was lower under the deception condition than under the truth telling condition in the high memory load (*p*< 0.001).

## Discussion

This study was designed to figure out the relationship between the information storage and processing in the construction component of deception, using CDA analysis. Consistent with the previous findings [33,41–43], we found that the CDA amplitude was significantly higher under high-load condition than under low-load condition. Moreover, a significant difference in the CDA amplitude was also found between deception and truth telling in the high-load tasks, while the low-load tasks did not show such difference. Further analysis found that the CDA amplitude was significantly suppressed by deception process in the high-load task.

The current study extended previous studies in at least three aspects. Firstly, to our best knowledge, this is the first deception study based on CDA, instead of P3 amplitude. Considering that P3 is associated with both the attention allocation and WM in the task [14,29], the CDA amplitude analysis could be directly modulated by the amount of information stored in memory, and exclude the potential interference of other cognitive processes. The results obtained in this study offer more convincing evidence for the thesis that WM is associated with the deception. Secondly, we manipulated two levels of WM load (4items for low-load task, and 6items for high-load task) during the deception process, by adopting the old/new recognition paradigm. The study could examine the difference in cognitive demand of deception between high-load and low-load conditions. Thus, it directly examined how the information storage and processing influenced WM allocation in the lie construction. Thirdly, in most deception research, participants should make a decision (to lie or not) and respond to the stimuli during the same test period. But in this study, participants knew whether to lie when they recognized the colored arrow, well before the presentation of the question. According to the ADCAT, we assured that the test period was mainly associated with the construction component. Thus, the results of the test period in this study mainly reflected the construction component of deception.

Because CDA amplitude could be used as an efficient index for the amount of information stored in memory, the change of the CDA amplitude between deception and truth telling could reveal the change of information storage during the lie construction in this study. The high-load (6-item) task showed a higher CDA amplitude than the low-load (4-item) task. This was consistent with the results of the WM capacity computation in the behavioral data analysis, and supported the claim that participants had the ability to keep more information in the WM when they had to remember more items during the high-load task [44,45]. The CDA amplitudes showed no significant difference between deception and truth telling in the low-load task, because the participants needed to remember 4 items both under these two conditions. Whereas, according to the behavioral data analysis, the computed WM capacity was significantly lower when the participants lied than told truth in the low-load task. We assumed that the behavioral performance was affected by many cognitive processes, such as recognition, attention, memory, inhibition. The low behavioral performance in deception tasks was due to the potential influence of the cognitive processes mentioned above. And many of these processes were excluded from the CDA analysis when we subtracted the ERP signals. The CDA amplitude remained stable in both the deception and truth telling response processes when task load was low, which revealed that the CDA amplitude was a better index for the WM capacity than the behavioral data, and the amount of information stored in WM was comparable when participants lied or told truth in the low-load tasks. Moreover, in the high-load tasks, we found that the CDA amplitude significantly decreased when participants lied, which was consistent with the results of the behavioral data analysis. This result showed that participants could keep more information in the WM when they gave honest response than when they deceived in high-load tasks. Previous studies revealed that participants needed to make up lies based on the truth-related information in the WM when they tried to lie[7,8], so that this construction process consumed the WM resources. Considering the limitation of the WM capacity, we assumed that the WM was insufficient for both the information maintenance and lie construction during the deception in the high-load tasks. Thus, the WM resources used to maintain information was consumed by the lie construction when participants lied in high workload, and participants could not store as much information in the WM as they did in the honesty tasks. As a result, the CDA amplitude decreased when participants lied in the high-load tasks. In contrast, in the low-load tasks, less information needed to be stored in WM, and thus more WM resources could be used for the following deception. The information storage was not affected by the information processing in the test period in the low-load task, and this was why the CDA amplitude showed no significant decrease when participants deceiving, compared with the truth telling.

The CDA results obtained in this study could better indicate that lie construction consumed WM resources, and the information storage and processing competed for the limited WM resources in the construction component. When only a few information was retrieved and maintained in WM for the incoming deception, the WM resources were sufficient for both information storage and processing during the lie construction. But if a large amount of information needed to be stored in WM for deception, WM might be insufficient for the lie construction. In this situation, when participants tried to give a deception response, lie construction would compete for extra WM resources and thus occupy the WM resources that information storage would have consumed. Thus, the information storage in the WM would be impaired by the lie construction during the deception process.

## Conclusion

In summary, this study manipulated the memory load during the deception task, and used the CDA amplitude as an efficient index to examine the relationship between WM and deception. This study found that the lie construction decreased the amount of information maintained in WM when participants lied in a high-load task, and it provided a directly evidence that supported the claim that WM participated in the deception process. Furthermore, the results revealed that WM played a role in the construction component of deception, which refined the previous claim. It could be assumed that the information storage and processing competed for the limited WM resources during the deception process. If there was too much information to store, the lie construction will occupy the WM resources demanded by the information storage, as the result of WM resource shortage, and the amount of information stored in WM decreased. But how cognitive system assigns WM resources to the information storage and processing during the lie construction still remains unclear. And we cannot examine the relationship between the WM and other deception components. In addition, because the CDA amplitude directly tracks the amount of stored information in the WM, it may play an important role in the deception researches. Considering the difference between the results of the CDA analysis and the capacity computation in behavioral data, we realize that an independent and efficient method is necessary to test individual’s total WM capacity, and this may make the results of the future study more reliable. It will be expectant that future studies will look into the deception, and focus on the cognitive process of each deception component. This may help us understand the inner cognitive mechanism of deception.
